# Supplementary material for: Global ocean methane emissions dominated by shallow coastal waters
Source: Nat Commun. 2019 Oct 8;10:4584. doi: 10.1038/s41467-019-12541-7 (PMC6783430; doi:10.1038/s41467-019-12541-7)
Supplement: Supplementary file 1 — Supplementary Information [file 41467_2019_12541_MOESM1_ESM.pdf]

Supplementary Information for Global ocean methane emissions dominated by shallow coastal waters, by Weber et al.

Supplementary Figures

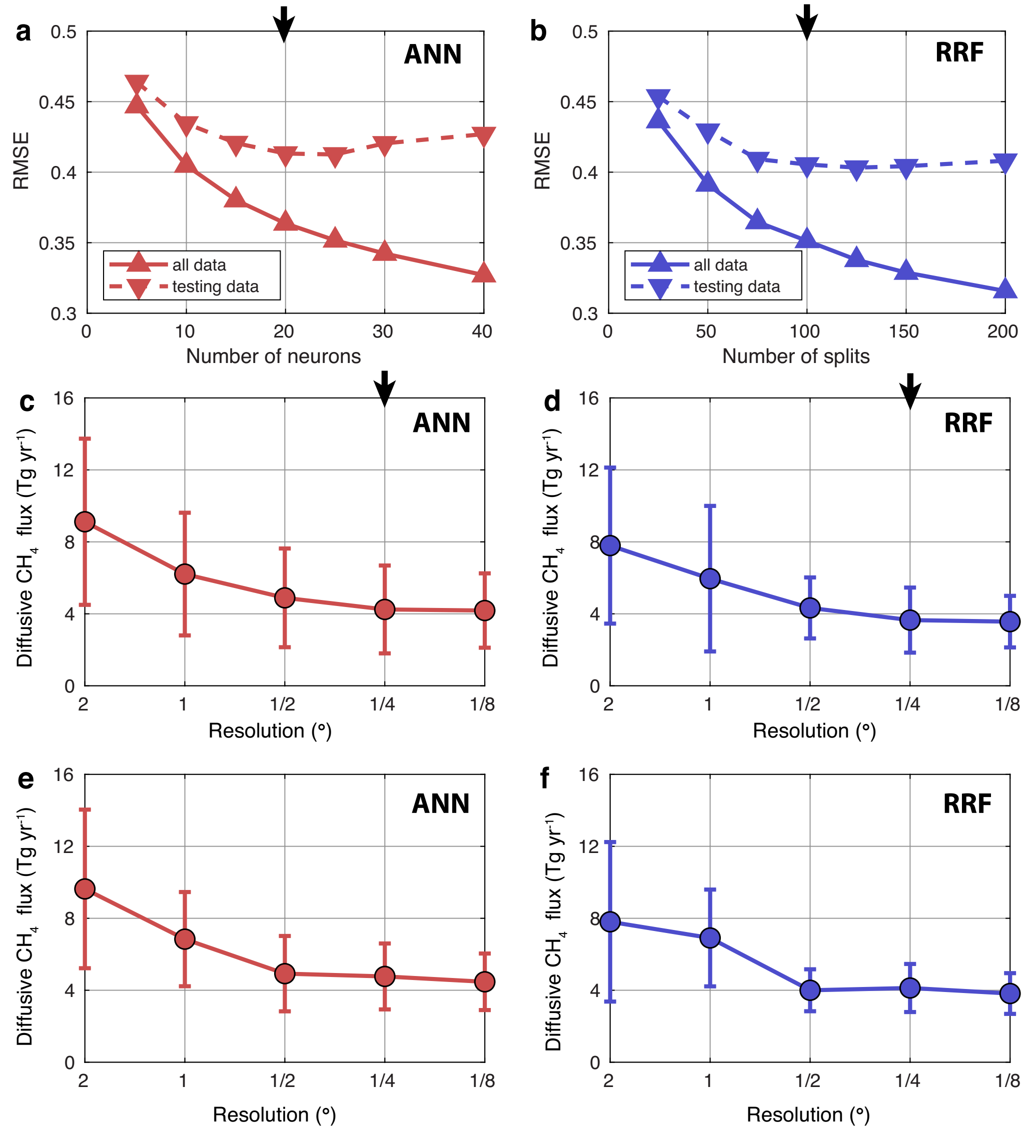

Supplementary Figure 1. Model sensitivity tests. a, Sensitivity of Artificial Neural Network (ANN) model skill (quantified as RMSE between observed and mapped transformed ΔCH_4) to the model complexity, defined as the number of neurons in the model's hidden layer. Improving the fit to the entire dataset, without improving the fit to testing data (not used in training the model) is a sign of overfitting. We therefore choose a level of complexity that maximizes model skill, while minimizing overfitting (black arrow). **b,** As **a** but for Random Regression Forest (RRF) method, where model complexity is defined as the maximum number of splits in a

regression tree. **c,** Sensitivity of global diffusive CH_4 emissions to the resolution of grid used for ANN mapping and flux calculations. **d,** Same as **c** but for RRF method. As grid resolution improves, the global flux eventually converges. For the results shown in the main text, we choose 0.25° resolution to achieve accurate flux estimates while maintaining computational efficiency. **e,f,** Same as **c-d** but for model ensembles that use chlorophyll-a instead of NPP as a predictor variable, which does not significantly impact the predicted fluxes or their dependence on grid resolution. In panels **c-f**, dots and error bars show the mean and standard deviation for 100 model runs at each grid resolution.

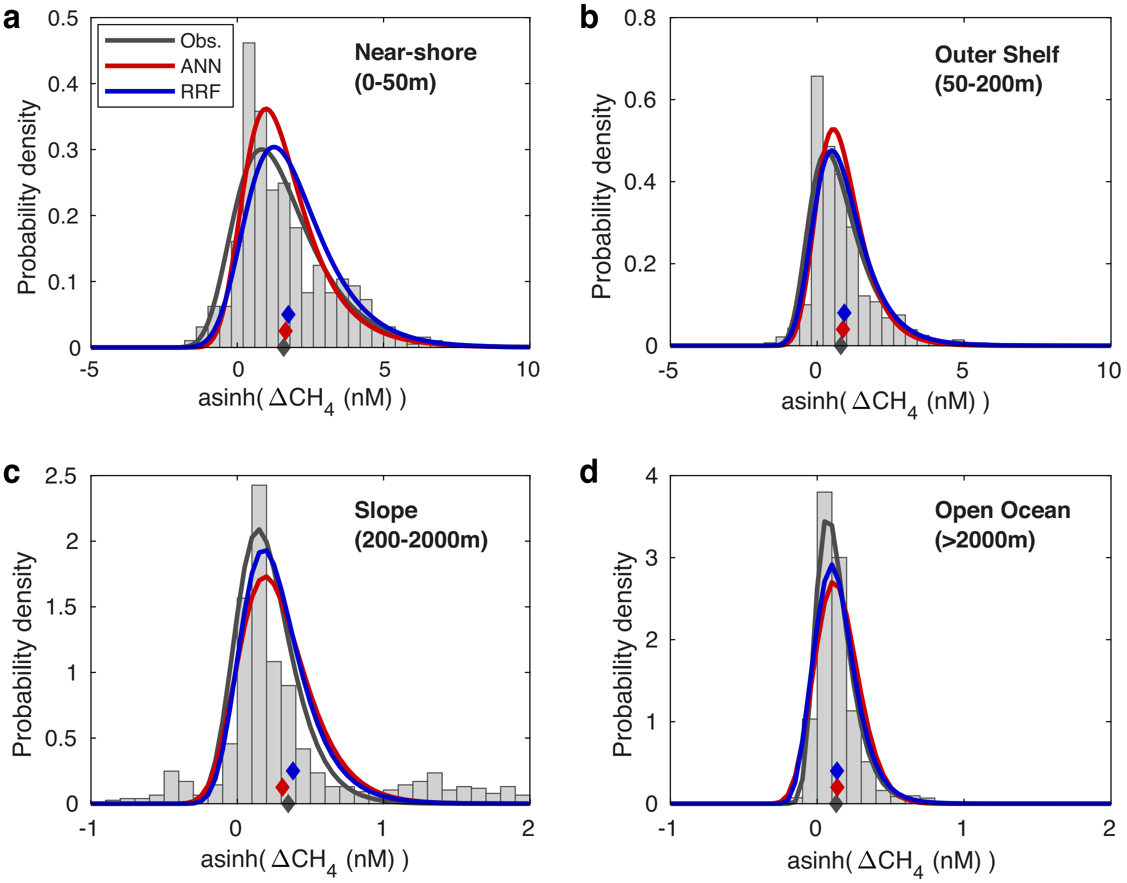

Supplementary Figure 2. Observed and modeled ΔCH_4 by region. Comparison between observed (grey bars and lines) and modeled (ANN=red lines; RRF=blue lines) ΔCH_4, after Inverse Hyperbolic Sine (IHS) transformation, in four bathymetric regions: (**a**) Near-shore, 0-50m; (**b**) Outer shelf, 50-200m; (**c**) Slope, 200-2000m; (**d**) Open ocean, >2000m. The IHS transformation is almost identical to a log transform but is defined for negative values of ΔCH_4. Continuous probability distributions (lines) are obtained by fitting a Generalized Extreme Value Distribution to the discrete data. Diamonds show the mean values from observations and the two mapping methods. Both methods closely reproduce the mean and spread of the observations in each region, and importantly accurately match the frequency of very high ΔCH_4 in near-shore and shelf environments (**a**,**b**), which drive a large portion of the global flux.

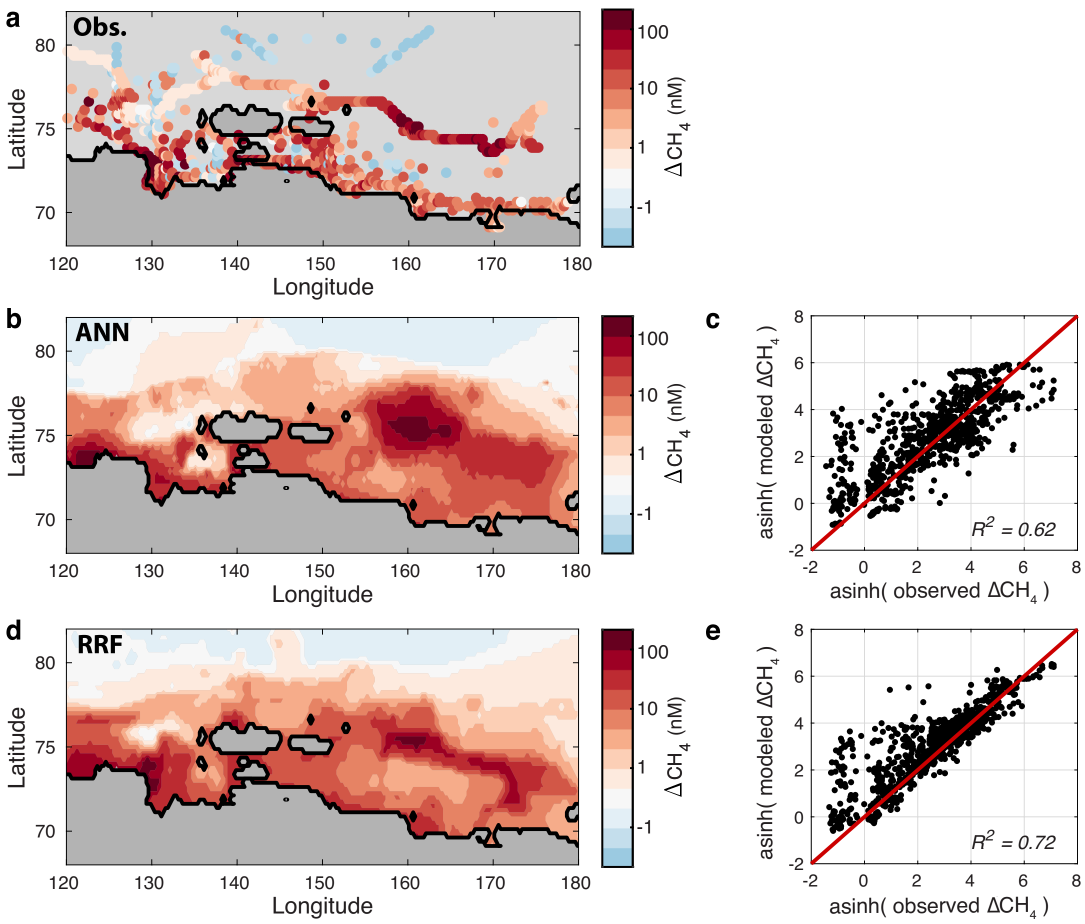

Supplementary Figure 3. ΔCH_4 in the ESAS region. a, Observed distribution of ΔCH_4 across the East Siberian Arctic Shelf (ESAS), averaged for August and September. **b,** Mapped August-September ΔCH_4 distribution from ANN method, averaged across all 100,000 ensemble members. **c,** Comparison between observed and ANN-mapped ΔCH_4 after IHS transformation. **d-e**, Same as **c-d** but for RRF method. Both mapping methods accurately reproduce observed ΔCH_4 in this region, including the frequency of very high supersaturations (especially RRF method, e). However, neither method predicts the extremely high diffusive emissions that have been inferred in this region from flux calculations based simple interpolation of the ΔCH_4 data [1] (Supplementary Table 1). This suggests that CH_4 emissions have previously been overestimated in this region.

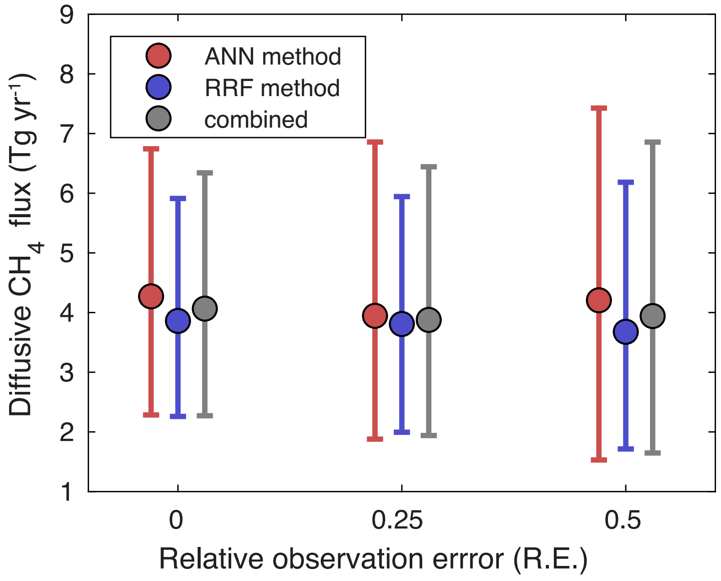

Supplementary Figure 4. Sensitivity to measurement error. Potential errors in [CH_4] measurements were propagated into ensembles of 100 ANN and RRF flux estimates, by generating synthetic datasets (see Methods) in which each [CH_4] datapoint diverges from the reported measured value by up 25% and 50% (Relative Error = 0.25, 0.5 respectively). This does not significantly impact the ensemble-mean fluxes (dots), but leads to modest expansion of the likely flux range (error bars show 10th-90th percentile range).

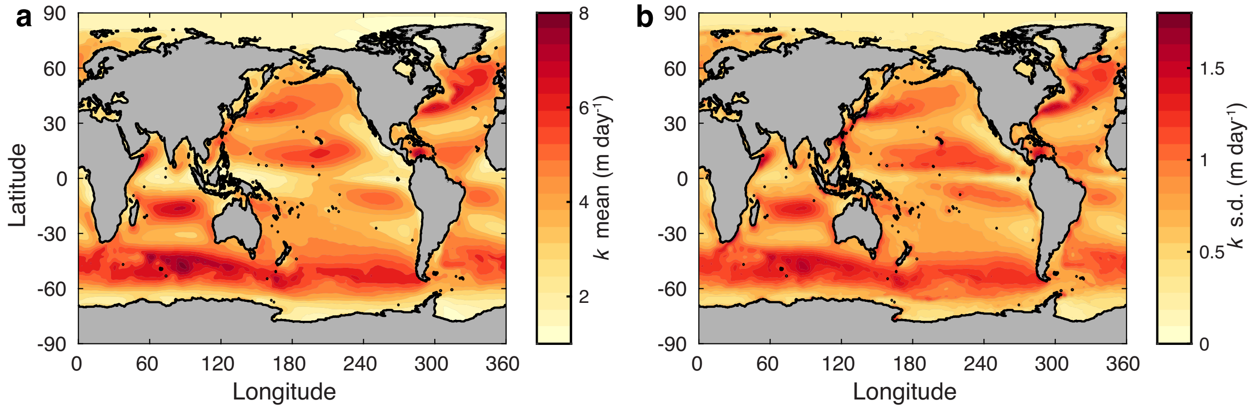

Supplementary Figure 5. Gas transfer velocity and uncertainty. a, Annual-mean gas transfer velocity (*k*), corrected for sea-ice inhibition, averaged across all permutations of four wind climatologies, three sea-ice climatologies, and five empirical algorithms relating *k* to wind speed (60 different estimates). **b,** Standard deviation in annual-mean k across the 60 different estimates. This uncertainty is propagated into our ocean-atmosphere flux calculations using a Monte Carlo procedure (see Methods).

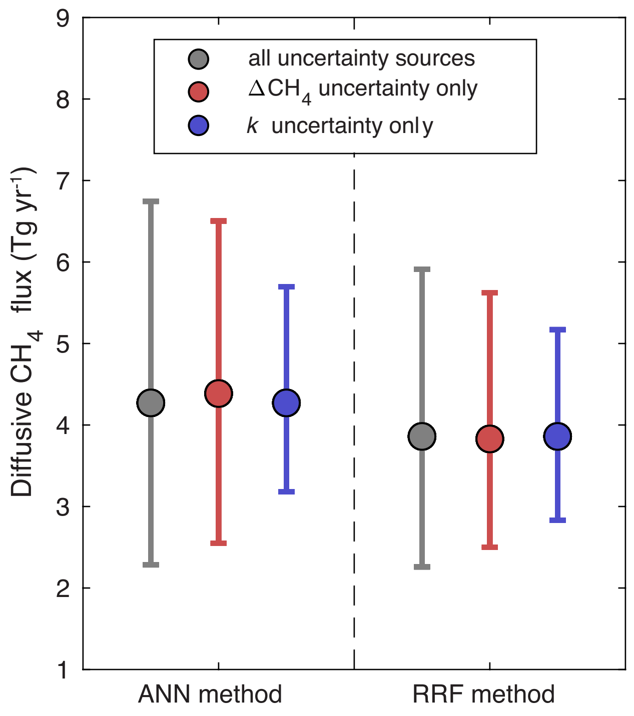

Supplementary Figure 6. Sources of uncertainty in global diffusive flux. We conducted two new suites global flux calculations to compare the contributions of uncertainty ΔCH_4 and k to uncertainty in global oceanic CH_4 emissions. In the first suite (red), 60 different mapped ΔCH_4 climatologies were generated from the ANN and RRF methods, and each was coupled with the same *k* climatology (average across 60 permutations) to isolate the effect of uncertainty in ΔCH_4. In the second suite (blue), the mean ΔCH_4 climatologies from our original ANN and RRF ensembles (Fig. 2a,b) were coupled with the 60 different *k* climatologies, to isolate the effect of uncertainty in *k*. Dots and error bars show the mean and 10th-90th percentile of the global flux in these suites. When only ΔCH_4 varies between iterations, the global flux was almost as uncertain as it was in our full ensemble (grey). In contrast, global flux was considerably (~40%) less uncertain when only *k* varies between iterations. This demonstrates that the ΔCH_4 distribution is the largest contributor to uncertainty in oceanic CH_4 emissions. This means that further constraining this flux can only be achieved by expanding the global dataset of ΔCH_4.

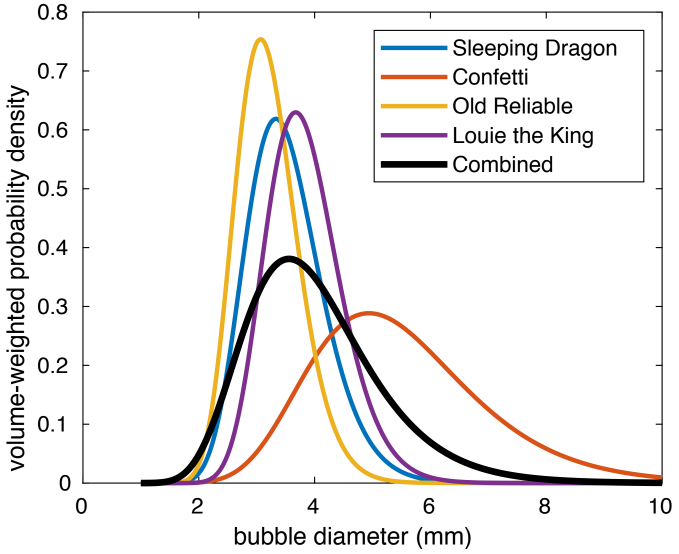

Supplementary Figure 7. Bubble size distribution at seafloor seeps. Colored lines show volume-weighted bubble size distributions observed at four different seep sites using high resolution imaging [2]. Thick black line represents a characteristic volume-weighted bubble size distribution, generated by combining equal numbers of samples from the four sites. This characteristic spectrum is used to estimate the transfer efficiency of CH_4 from the seafloor to ocean surface in rising bubbles (Fig. 5a).

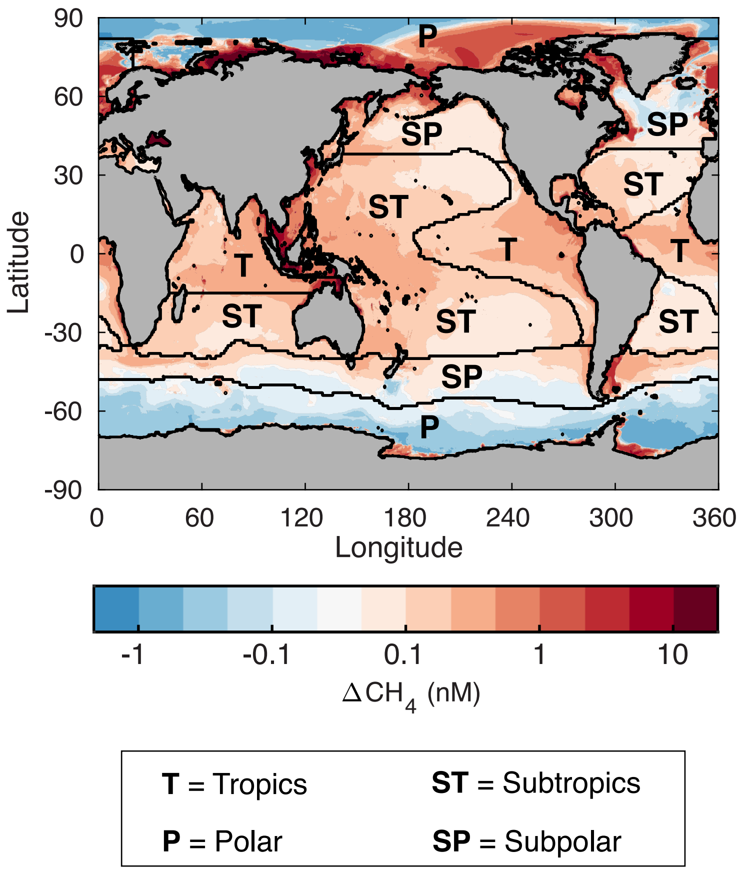

Supplementary Figure 8. Open-ocean pattern of ΔCH_4. Color map is the annual mean ΔCH_4 distribution averaged across all ANN and RRF ensemble members (i.e. the average of Fig. 2a-b). This distribution is correlated against predictor variables to assess controls on methane supersaturation (Supplementary Table 2, see Methods). Furthermore, to assess the large-scale latitudinal pattern, we take broad averages across Tropical, Subtropical, Subpolar, and Polar regions, separated by the black contour lines. These regions are defined as in ref. 3, and the Indian Ocean is separated into Tropics and Subtropics along 15°S.

Supplementary Tables

Supplementary Table 1. Ensemble-mean ΔCH_4 and flux by ocean basin. Mean ΔCH_4 and integrated ocean-atmosphere flux were computed from ensemble-mean climatologies for each major ocean basin. Properties are further divided into regions shallower than 200m (near-shore and outer shelf environments) and those deeper than 200m (continental slope and open ocean environments).

Basin	Mean ΔCH_4 (nM)	Ocean-Atmosphere flux (Tg yr^{-1})
Atlantic	0.86	1.00
<200m	7.63	0.77
>200m	0.19	0.23
Pacific	0.76	1.84
<200m	10.01	1.19
>200m	0.24	0.65
Indian	0.56	0.48
<200m	5.89	0.23
>200m	0.23	0.25
Southern	0.04	0.06
<200m	3.81	0.08
>200m	-0.03	-0.02
Arctic	6.73	0.48
<200m	18.5	0.41
>200m	0.88	0.05

Supplementary Table 2. Statistical analysis of ΔCH_4 distribution. R^2 statistic for linear regression between ensemble-mean mapped ΔCH_4 and each predictor variable for coastal ocean (<2000m depth) and open ocean (>2000m depth) environments. The signs in parentheses indicate whether there is a positive or negative correlation between the two.

Predictor variable	R^2 (direction)	
	Coastal ocean (<2000m), against $\log_{10}(\Delta CH_4)$	Open ocean (>2000m), against ΔCH_4
Seafloor depth	0.366 (-)	2×10^{-5} (-)
$\log_{10}$(Seafloor depth)	0.548 (-)	5×10^{-4} (-)
Net Primary Production (NPP)	0.018 (+)	0.295 (+)
POC export flux	0.108 (+)	0.142 (+)
$[PO_4]$	0.001 (-)	0.173 (-)
Temperature	3×10^{-6} (+)	0.221 (+)
Salinity	0.104 (-)	0.006 (+)
Subsurface $[O_2]$	0.077 (+)	0.157 (-)
Sediment hydrate inventory	0.028 (-)	0.078 (+)
Dimethyl sulfide (DMS)	3×10^{-4} (-)	0.042 (+)

References

1 Shakhova, N. *et al.* Extensive Methane Venting to the Atmosphere from Sediments of the East Siberian Arctic Shelf. *Science* **327**, 1246-1250 (2010).

2 Wang, B., Socolofsky, S. A., Breier, J. A. & Seewald, J. S. Observations of bubbles in natural seep flares at MC 118 and GC 600 using in situ quantitative imaging. *Journal of Geophysical Research: Oceans* **121**, 2203-2230, doi:10.1002/2015jc011452 (2016).

3 Weber, T., Cram, J. A., Leung, S. W., DeVries, T. & Deutsch, C. Deep ocean nutrients imply large latitudinal variation in particle transfer efficiency. *Proceedings of the National Academy of Sciences*, 201604414 (2016).
